# Tax1BP1 limits hepatic inflammation and reduces experimental hepatocarcinogenesis

**DOI:** 10.1038/s41598-020-73387-4

**Published:** 2020-10-01

**Authors:** Oliver Waidmann, Thomas Pleli, Andreas Weigert, Esther Imelmann, Bianca Kakoschky, Christian Schmithals, Claudia Döring, Matthias Frank, Thomas Longerich, Verena Köberle, Martin-Leo Hansmann, Bernhard Brüne, Stefan Zeuzem, Albrecht Piiper, Ivan Dikic

**Affiliations:** 1 Medizinische Klinik 1, Schwerpunkt Gastroenterologie Und Hepatologie, Universitätsklinikum Frankfurt, Goethe-Universität, Theodor-Stern-Kai 7, 60590 Frankfurt, Germany; 2 Institut für Biochemie 2, Universitätsklinikum Frankfurt, Goethe-Universität, Theodor-Stern-Kai 7, 60590 Frankfurt, Germany; 3 Institut für Biochemie 1, Universitätsklinikum Frankfurt, Goethe-Universität, Theodor-Stern-Kai 7, 60590 Frankfurt, Germany; 4 Senckenbergsches Institut für Pathologie, Universitätsklinikum Frankfurt, Goethe-Universität, Theodor-Stern-Kai 7, 60590 Frankfurt, Germany; 5 Sektion Translationale Gastrointestinale Pathologie, Institut für Pathologie, Universitätsklinikum Heidelberg, Im Neuenheimer Feld 224, 69120 Heidelberg, Germany

**Keywords:** Cancer, Immunology

## Abstract

The nuclear factor kappa beta (NFκB) signaling pathway plays an important role in liver homeostasis and cancer development. Tax1-binding protein 1 (Tax1BP1) is a regulator of the NFκB signaling pathway, but its role in the liver and hepatocellular carcinoma (HCC) is presently unknown. Here we investigated the role of Tax1BP1 in liver cells and murine models of HCC and liver fibrosis. We applied the diethylnitrosamine (DEN) model of experimental hepatocarcinogenesis in Tax1BP1^+/+^ and Tax1BP1^−/−^ mice. The amount and subsets of non-parenchymal liver cells in in Tax1BP1^+/+^ and Tax1BP1^−/−^ mice were determined and activation of NFκB and stress induced signaling pathways were assessed. Differential expression of mRNA and miRNA was determined. Tax1BP1^−/−^ mice showed increased numbers of inflammatory cells in the liver. Furthermore, a sustained activation of the NFκB signaling pathway was found in hepatocytes as well as increased transcription of proinflammatory cytokines in isolated Kupffer cells from Tax1BP1^−/−^ mice. Several differentially expressed mRNAs and miRNAs in livers of Tax1BP1^−/−^ mice were found, which are regulators of inflammation or are involved in cancer development or progression. Furthermore, Tax1BP1^−/−^ mice developed more HCCs than their Tax1BP1^+/+^ littermates. We conclude that Tax1BP1 protects from liver cancer development by limiting proinflammatory signaling.

## Introduction

Hepatocellular carcinoma (HCC) is an increasing burden in the Western world and it is the third most common cause for cancer mortality worldwide^1,2^. The majority of HCC cases develop due to chronic tissue damage resulting from chronic hepatitis B virus (HBV) or hepatitis C virus (HCV) infections, alcohol abuse, fatty liver disease or genetic reasons such as hemochromatosis.

The NFκB as well as other signaling pathways, including the mitogen-activated protein kinases (MAPKs) and the mammalian target of rapamycin (mTOR) signaling pathway, play important roles in HCC development and progression^3–5^. Whereas inhibition of NFκB by an IκBα super-repressor can impede tumor progression in MDR2 knock-out mice, which develop liver cancer as a result of chronic cholangitis^6^, a complete loss of the central NFκB activator IKKγ/NEMO in hepatocytes leads to spontaneous liver cancer development^7^. Furthermore, a loss of the catalytical IκB kinase β (IKK β) in hepatocytes results in enhanced tumor formation in a mouse model of chemical induced HCC^8^.

There are five NFκB proteins (RelA/p65, RelB, c-Rel, p100, p105), which form dimers and are retained in the cytoplasm by the inhibitory IκB proteins^9^. After activation of the central IKK (IκB kinase) complex by signaling cascades resulting from activation of transmembrane receptors by proinflammatory cytokines, such as tumor necrosis factor α (TNFα), bacterial lipopolysaccharides (LPS) or interleukin-1β (IL-1β), the inhibitory proteins are phosphorylated and ubiquitinated and thereby marked for proteasomal degradation. As soon as the dimers are released, they translocate to the nucleus, bind to NFκB binding sites and regulate the transcription of proinflammatory and anti-apoptotic genes^9^. Beyond the anti-apoptotic signals, which are important not only in the liver but also in the colon for maintaining homeostasis^10^, the loss or reduced expression of negative regulators of NFκB such as the deubiquitinating protein CYLD in has been found in HCC, and genetic ablation of CYLD increases hepatocarcinogenesis^11,12^. In addition, myeloid cells play an important role in cancer progression by production of inflammatory cytokines, which activate NFκB and thereby support cancer progression^3^. Thus, activation of NFkB signaling is a key cancer-promoting mechanism.

An important regulator of the NFκB signaling pathway is Tax1BP1. Initially, this protein has been characterized as a binding partner of TRAF6 and A20, which are involved in the activation and inhibition of the NFκB signaling pathway, respectively^13,14^. Tax1BP1 has been identified as an ubiquitin binding protein that acts as a negative regulator of TNF-α and IL-1β induced NFκB activation by recruiting the deubiquitinating enzyme A20 to activated receptor complexes^15^. It cooperates in a complex with A20 and ITCH in inhibition of the NFκB pathway downstream of TNFα and LPS^15,16^. Loss of Tax1BP1 in mice leads to increased inflammation in different organs, including the liver, with upregulation of NFκB signaling^15^. Transplantation of bone marrow from wild type to Tax1BP1^−/−^ mice can reverse the inflammatory phenotype, indicating that the inflammatory phenotype depends on myeloid derived cells^15^. Chronic inflammation in liver diseases is regulated and promoted by non-parenchymal liver cells, most notably resident and infiltrating bone marrow-derived macrophages and hepatic stellate cells^3^. Tax1BP1^−/−^ mice show an increase in the number of macrophages in the liver, implying higher inflammatory activity^17^. Furthermore, a gain of Tax1BP1 genomic copy number in a subset of HCCs has been described^18^. Nevertheless, the role of Tax1BP1 in liver cancer development is still unknown. In the present study we further characterized the role of Tax1BP1 in liver homeostasis and carcinogenesis in Tax1BP1^−/−^ and wild type mice by applying tissue microarrays, isolation and characterization of hepatocytes and primary leukocytes as well as the DEN model of hepatocarcinogenesis.

## Materials and methods

### Animals

Tax1BP1^−/−^ mice which have been backcrossed to a C57/BL6 background have been described previously^15^. The mice were housed under 12 h day/night cycles, were fed standard rodent chow and had access to drinking water ad libitum. All animal experiments were approved by the Regierungspräsidium Darmstadt of the state Hessen, Germany (V54—19 c 20/15—F142/02 and V54—19 c 20/15—FK/1010). All experiments were performed in accordance with relevant guidelines and regulations.

### Extraction of genomic DNA und genotyping PCR

Mouse tissue was suspended in a Tris-buffer and was digested with proteinase K for one hour at 55 °C. The genotype was assessed by PCR with the primer pairs described before^15^.

### Isolation and culture of mouse hepatocytes

Murine hepatocytes were isolated and cultured according to Klingmüller et al.^19^ with slight modifications. Mice were anesthetized with Ketamin (100 mg/kg body weight) and Xylazin (10 mg/kg body weight). After careful disinfection the animals were laparotomized and the inferior vena cava was identified. The vein was punctured and a 22 gauge venous catheter was placed. After dissection of the portal vein the liver was perfused retrogradely with a buffer warmed up to 37 °C containing Hank’s buffered saline (HBSS), 15 mM HEPES, 2.5 mM EGTA, glucose (1 g/l) and penicillin and streptomycin for 10 min and at a flow rate of 10 ml/min. Then the liver was perfused with a second buffer containing HBSS, 15 mM HEPES, 5 mM calcium chloride and collagenase IV (25 U/ml) for 10 min and at a flow rate of 10 ml/min. The liver was excised, transferred to a petri dish, carefully dissected and resuspended in 10 ml Williams Medium E containing 10% fetal calf serum (FCS). The suspension was carefully flushed through a cell strainer (100 mm; BD Biosciences, Heidelberg, Germany) and transferred to a 50 ml tube. The tube was filled up to 50 ml with medium and a centrifugation step of 5 min with 50×*g* at 4 °C was performed. The supernatant was discarded and the cells were resuspended in 50 ml of fresh medium, followed by an additional centrifugation step at 4 °C for 5 min. The supernatant was again discarded and the cells were once again resuspended in 10 ml medium. The number of viable cells was assessed with trypan blue staining. 10^6^ viable cells were seeded in 2 ml medium in each well of collagen coated six well plates. After 2 h the supernatant was removed and the attached cells were washed with phosphate buffered saline (PBS) and covered with 2 ml fresh medium containing 10% FCS. On next day the hepatocytes were stimulated with murine TNFα (10 ng/ml, Peprotech GmbH, Hamburg, Germany) for the indicated time.

### Isolation and culture of mouse Kupffer cells

Kupffer cells were isolated according to Froh et al.^20^ with slight modifications. For isolation of Kupffer cells, the livers of anesthetized mice were perfused as described above. After resuspension of the liver homogenate in 10 ml Williams Medium E containing 10% FCS, the suspension was centrifuged 50×*g* at 4 °C for 5 min. The supernatant was collected and transferred to a new 50 ml tube and a second centrifugation step with 50×*g* at 4 °C for 5 min was performed. The supernatant obtained at the second step was centrifuged for at 650×*g* at 4 °C for 7 min. The pellet was resuspended in 1 ml HBSS and added to a 15 ml tube, which was overlayed with 7 ml layers of 50% and 25% Percoll, respectively. The tube was centrifuged at 1800×*g* and at 4 °C for 15 min. The medium layer of the newly formed solution was transferred to a new 15 ml tube and filled up to 15 ml with HBSS. After an additional centrifugation step at 650×*g* and at 4 °C for 7 min, the pellet was resuspended in 1 ml HBSS. After cell counting, the macrophages were isolated with anti F80/4-biotin labelled antibodies using magnetic activated cell sorting (MACS) (Miltenyi Biotec GmbH, Bergisch Gladbach, Germany) according to the recommendation of the manufacturer. Isolated Kupffer cells were resuspended in RPMI 1640 medium containing 10% FCS and 1% penicillin/streptomycin and were plated on 6 well plates. After 1 h the medium was changed. On the next day the Kupffer cells were stimulated with LPS (300 ng/ml, InvivoGen, Toulouse, France) for the indicated time points.

### Isolation and characterization of hepatic leucocytes by flow cytometry

Characterization of immune cell subsets in the liver was performed essentially as described previously^21^. The individual samples were analyzed with a LSRII/Fortessa flow cytometer (BD Biosciences) and the FlowJo software Vx (Treestar). All indicated antibodies and reagents were titrated to determine optimal concentrations. CompBeads (BD) were used for single-color compensation to create multi-color compensation matrices. For gating, fluorescence minus one controls were used. The instrument calibration was controlled daily using cytometer setup and tracking beads (BD). Single cell suspensions were created using the Miltenyi Liver Dissociation Kit (No. 130-105-807) and the GentleMACS isolator (Miltenyi) using standard protocols. The following antibodies were used: anti-CD3-PE-CF594, anti-CD4-V500, anti-CD11c-AlexaFluor700, anti-CD19-APC-H7, anti-CD326 (EPCAM)-BV711, anti-Ly6C-PerCP-Cy5.5 (all from BD), anti-CD8-eFluor650, anti-CD11b-eFluor605NC (eBioscience), anti-CD45-VioBlue, anti-CD49b-PE, anti-MHC-II-APC (Miltenyi), anti-F4/80-PE-Cy7, anti-Ly6G-APC-Cy7 (Biolegend). A gating strategy is provided in the supplementary material and methods (Fig. S1).

### DEN induced tumor induction

For induction of HCC male mice were injected at day 15 of life with 25 mg/kg DEN (Diethylnitrosamine, Sigma-Aldrich Chemie GmbH, Taufkirchen, Germany)^8^. After eight months the mice were sacrificed, the livers were removed, fixed in formalin and subjected to pathological examination. In detail, the right medial lobe was cut in sections of two millimeter and two hematoxylin and eosin stained slides from every section were used for determination of the number of nodules for every mouse, respectively.

### Fibrosis induction with CCl_4_

Female and male mice aged 7–8 weeks received intraperitoneal injections of 0.375 µg/g CCl_4_ (Sigma-Aldrich Chemie GmbH, Taufkirchen, Germany) dissolved in olive oil twice weekly for six following weeks. One week after last injection, the mice were sacrificed. Liver were excised and were fixed in buffered formalin. For fibrosis evaluation four different sections at different depths through the left liver lobe were performed and were evaluated for fibrosis development.

### Confocal microscopy of murine liver tissue sections

Tax1BP1^+/+^ mice were sacrificed and the liver tissue was fixed with 4% paraformaldehyde for 24 h. Ten micrometer-cryosections were prepared, rehydrated in PBS and blocked with 2% bovine serum albumin (BSA)/PBS solution containing 0.1% Triton X-100. The tissue was incubated with rabbit anti-TRAF6BP (= Tax1BP1; 1:100 dilution; Abcam) and with rat anti-mouse CD68 (1:100 dilution; Biolegend) in PBS containing 2% BSA and 0.1% Triton X-100 overnight at 4 °C. After washing with PBS, the sections were further incubated with Alexa Fluor 546 conjugated goat anti-rabbit (1:200 dilution; Invitrogen) and Alexa Fluor 633 conjugated goat anti-rat (1:200 dilution; Invitrogen) for 1 h at room temperature. Both secondary antibodies were diluted in PBS containing 2% BSA and 0.1% Triton X-100. Sections without rabbit anti-TRAF6BP staining were used as negative controls. All coverslips were mounted on slides with Permount toluene solution (Fisher Chemicals) and imaged using an Olympus Fluoview 1000 confocal microscope.

### Histochemistry and immunohistochemistry

Murine hepatic tissue samples were formalin fixed and paraffin embedded and cut at a thickness of 4 µm. After dewaxing and rehydration the slides were stained with hematoxylin and eosin for standard histochemistry and/or Sirius red staining for fibrosis staining. For immunhistochemistry the slides were deparaffinized and rehydrated. Then slides were incubated with PBS containing 3% BSA for 60 min. Primary antibodies were added for 60 min at room temperature and after washing several times the biotinylated secondary antibodies were added for 30 min. After several additional washing steps with PBS, the tissue slides were incubated for 30 min with streptavidin peroxidase and the development reaction with the DAB chromogen was performed for 5–15 min.

### mRNA extraction and real-time PCR

Total RNA from mouse livers was extracted with the high pure mRNA extraction kit from Roche. cDNA was generated with random primers. Real-time PCR was performed on an Applied Biosystems StepOnePlus system with commercially available TaqMan Copy Number Assays for Tax1BP1, IL-1β, IL-6, TNFα, and Glycerol-3-phosphate dehydrogenase (GPDH) (Thermo Fisher Scientific Inc., Waltham, Massachusetts, USA). Expression of Tax1BP1, IL-1β, IL-6, and TNFα were normalized to GPDH expression.

### Western blotting

Western blotting was performed as described previously^22^. The membranes were incubated with the SuperSignal West Pico Chemiluminescent Substrate from Thermo Scientific (Rockford, IL) and the signals were detected by the Fuji LAS-4000 detection system. The following primary antibodies were used: mouse (1:500) and rabbit (1:500) Tax1BP1 and mouse β-actin antibodies (1:5000) were from Sigma-Aldrich Chemie GmbH, Taufkirchen, Germany. Mouse pSAPK/JNK (Thr183/Tyr185) (1:500), rabbit SAPK/JNK (1:1000), mouse IκBα (1:1000), mouse phospho-NF-kappa-B p65 (Ser536) (1:500), and rabbit phospho-NF-kappa-B p65 (1:1000) antibodies were purchased from Cell Signaling Technologies, Danvers, MA, USA. For quantification of Western Blot signals Image J software (National Institutes of Health, Bethesda, MD, USA). The mean value of three independent experiments was calculated and is shown in the figure.

### Gene and miRNA arrays

Materials and methods including analyses are reported in Supplementary File 1. The data discussed in this publication have been deposited in the NCBI Gene Expression Omnibus and are accessible through GEO series accession numbers GSE98908 for the gene array and GSE98907 for the miRNA array, respectively.

### Statistical analysis

Statistically analyses were performed with BIAS 10.04 for Windows software (Epsilon Verlag, Darmstadt, Germany). Differences between groups were assessed with the two sided t-test. P values < 0.05 were considered to be significant. Values were tested for normal distribution. If no normal distribution was found, Welch's t-Test was applied. In the column blots the mean values and the standard deviation are shown.

## Results

### Tax1BP1 expression in murine liver

**Figure 1:**
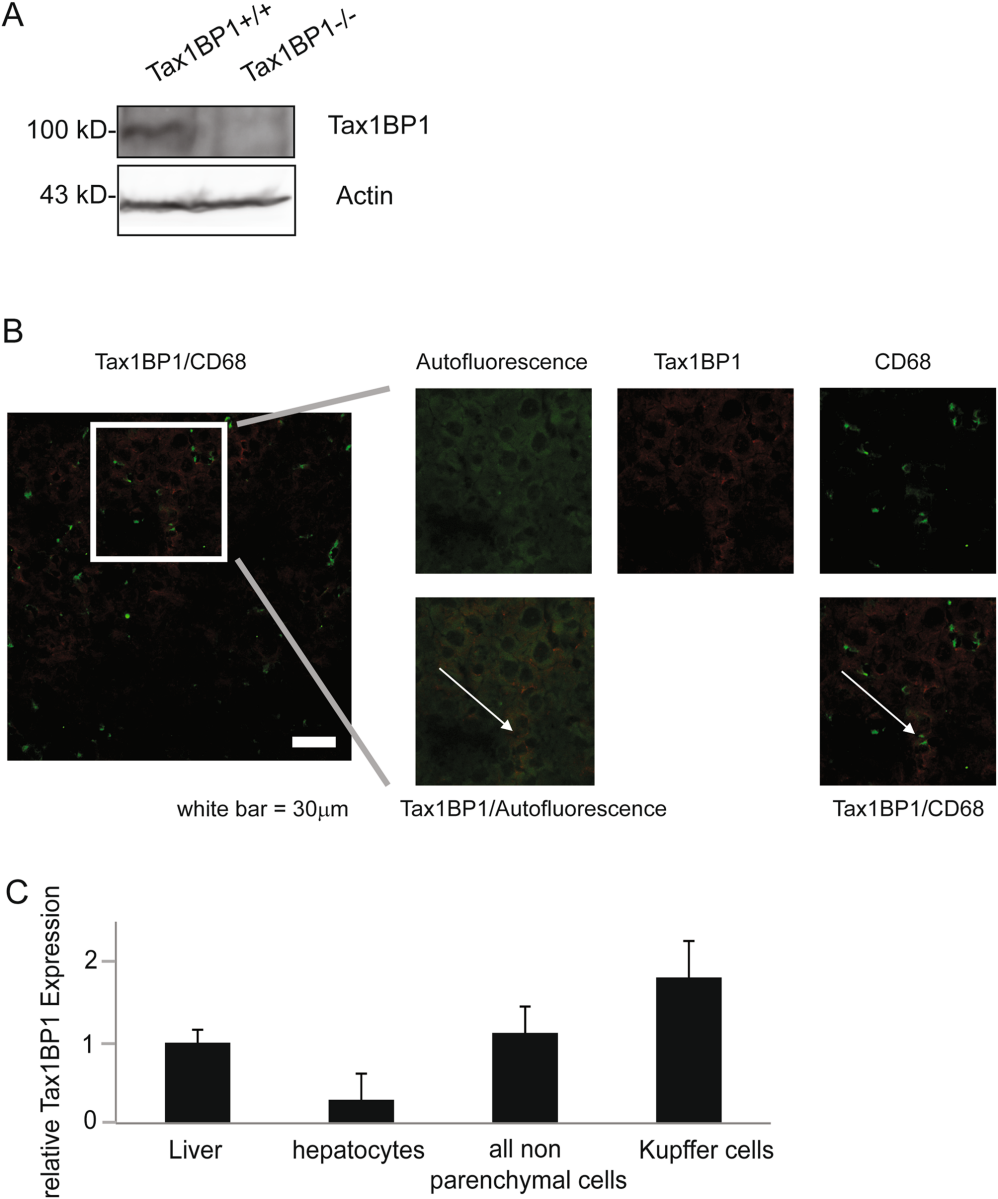
Tax1BP1 expression in different hepatic compartments. Tax1BP1 expression was determined in liver lysates of Tax1BP1^+/+^ and Tax1BP1^−/−^ mice by Western Blotting using a polyclonal antibody (**A**). Confocal laser scanning microscopy showing hepatocytes´ autofluorescence (green), Tax1BP1 (red), and CD68 (green) (magnification × 400). Arrows in the cutouts show representative areas of colocalization of the indicated stainings, respectively (**B**). Quantitative PCR was performed to assess relative Tax1BP1 mRNA expression, normalized to GPDH, in whole liver, hepatocytes, all non-parenchymal cells and Kupffer cells. Relative Tax1BP1 expression is shown in relation to whole liver (**C**).

Female and male Tax1BP1^+/−^ mice were crossed to obtain Tax1BP1^+/+^, Tax1BP1^+/−^ and Tax1BP1^−/−^ mice. The mice were genotyped with primer pairs as described previously^15^. Tax1BP1 is widely expressed in different organs including liver tissue^23^. Western blotting analysis of the livers from Tax1BP1^+/+^ and Tax1BP1^−/−^ mice revealed Tax1BP1 expression in Tax1BP1^+/+^ and absence of its expression in Tax1BP1^−/−^ mice (Fig. 1A). To assess the expression pattern of Tax1BP1 in parenchymal and non-parenchymal cells, confocal microscopy of murine liver tissue sections from Tax1BP1^+/+^ mice was performed. Tax1BP1 expression could be detected in non-parenchymal cells as well as in hepatocytes (Fig. 1B). To further analyze Tax1BP1 expression in different hepatic cell compartments, quantitative PCR of Tax1BP1 mRNA was performed in the fraction of isolated hepatocytes as well as in the fraction of non-parenchymal cells. Additionally, quantitative PCR of Tax1BP1 mRNA was performed in isolated Kupffer cells. As shown in Fig. 1C non-parenchymal cells, and particular Kupffer cells, showed a higher Tax1BP1 expression than hepatocytes.

### Increased number of inflammatory cells and sustained inflammatory responses of macrophages in livers of Tax1BP1^−/−^ mice

**Figure 2:**
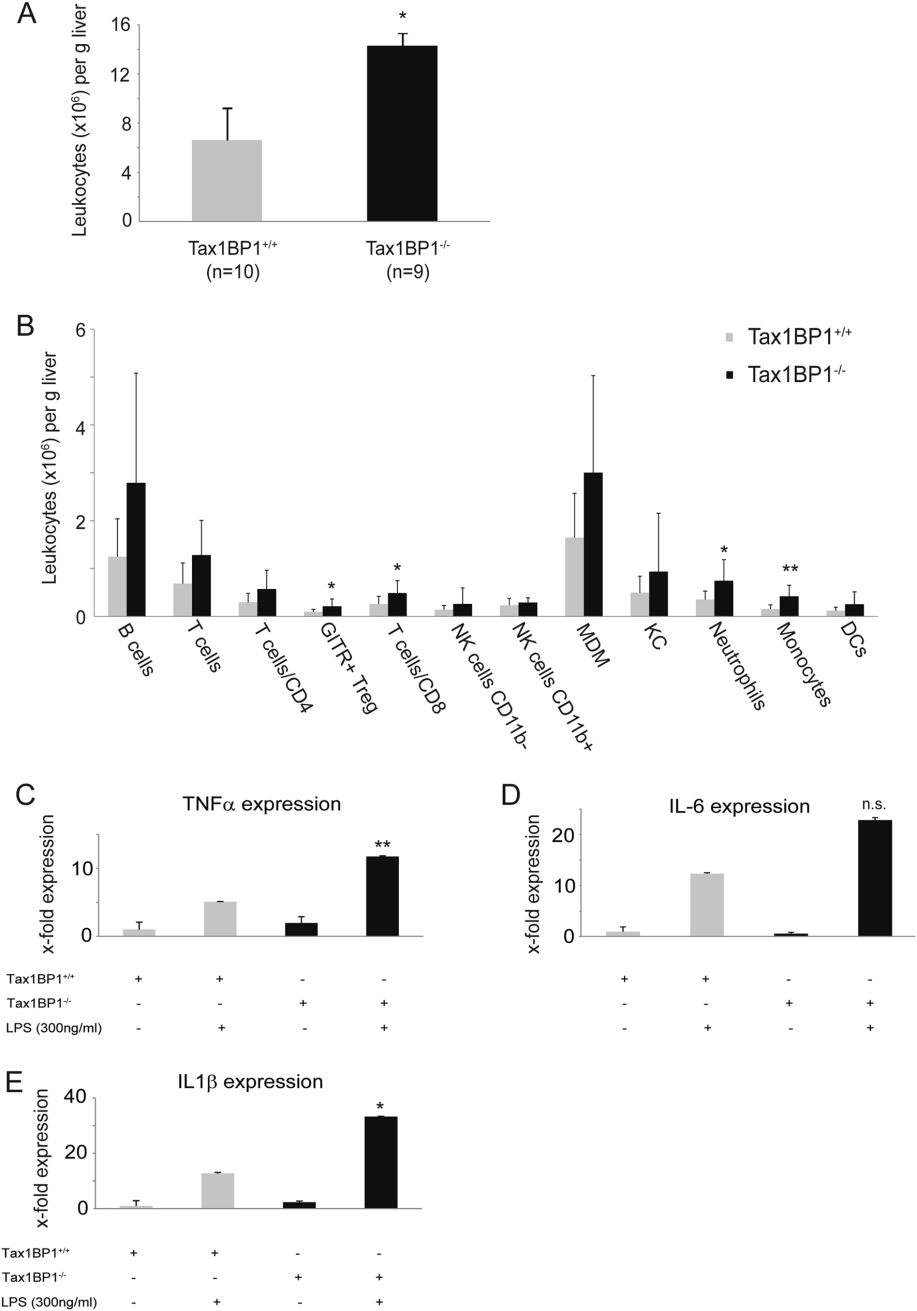
Increased number of leukocytes and increased inflammatory responses in Tax1BP1^−/−^ mice. Number of all leukocytes (**A**) and different subpopulations of leukocytes (**B**) were determined by FACS from livers of Tax1BP1^+/+^ and Tax1BP1^−/−^ mice, respectively. mRNA of TNFα, IL-6 and IL1β was determined by quantitative PCR in Kupffer cells extracted from two livers of Tax1BP1^+/+^ and Tax1BP1^−/−^ mice ± stimulation with LPS 300 ng/ml for 6 h. Relative expression of mRNA was normalized to unstimulated Tax1BP1^+/+^ mice. Representative results of two independent experiments are shown (**C**–**E**). (*P < 0.05; **P < 0.01; *GITR* glucocorticoid-induced tumor necrosis factor receptor related protein, *NK cells* natural killer cells, *MDM* monocyte-derived macrophages, *KC* Kupffer cells, *DC* dentritic cell).

Increased numbers of macrophages in livers of Tax1BP1^−/−^ mice were described previously^17^. However, a more detailed characterization of the innate and adaptive immune cells has not been performed in Tax1BP1^−/−^ mice. Therefore, hepatic leukocyte patterns from terminally perfused mice were characterized. FACS analysis of isolated leukocytes of Tax1BP1^+/+^ and Tax1BP1^−/−^ mice revealed elevated numbers of leukocytes in livers of Tax1BP1^−/−^ mice (*P* = 0.028) (Fig. 2A). Further FACS analysis of suspended liver cells revealed increased levels of immune cells of the innate as well as adaptive immune system in Tax1BP1^−/−^ mice (Fig. 2B). Especially, increases in regulatory T cells, CD8 positive T cells, neutrophils and monocytes were statistically significant (Fig. 2B). To further characterize innate inflammatory responses in Tax1BP1^−/−^ mice compared to Tax1BP1^+/+^ mice, Kupffer cells from the indicated mice were isolated and stimulated with LPS. As shown in Fig. 2C–E Kupffer cells from Tax1BP1^−/−^ mice showed a significantly higher transcription mRNA of TNFα and IL1β compared to their wildtype littermates. The transcription of IL-6 mRNA was also increased. However, the increase was not statistically significant.

### Loss of Tax1BP1 leads to sustained NFκB activation in hepatocytes

**Figure 3:**
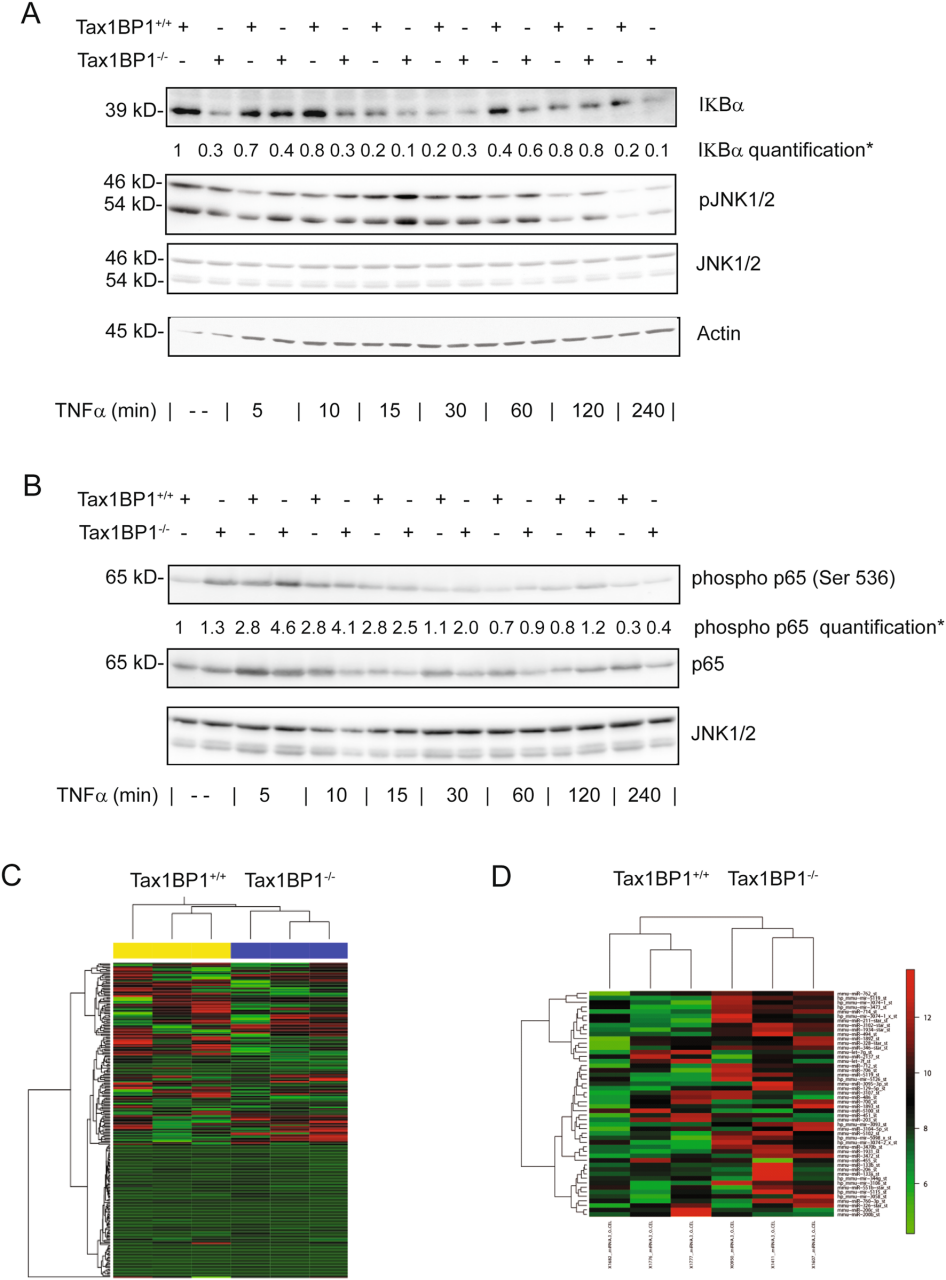
Activation of NFκB and JNK pathways in hepatocytes and differentially regulated mRNA and miRNA in Tax1BP1^+/+^ and Tax1BP1^−/−^ mice. Livers of mice were extracted after terminal perfusion and hepatocytes were isolated. Hepatocytes were stimulated with TNFα for the indicated time points and Western Blotting was performed with the indicated antibodies. Representative results of three independent isolations of primary cells are shown. Densitometry was performed for three independent blots and mean values are shown (*) (**A**,**B**). Livers form three Tax1BP1^+/+^ and three Tax1BP1^−/−^ mice were excised, RNA was extracted and microarray analyses were performed determining mRNA (**C**) and miRNA (**D**) expression.

As Tax1BP1 expression was found in parenchymal as well as in non-parenchymal cells and Tax1BP1^−/−^ mice showed an increase in hepatic leukocytes and increased production of proinflammatory cytokines following stimulation with LPS, we investigated if hepatocytes from Tax1BP1^−/−^ mice show increased NFκB activation. Therefore, hepatocytes were isolated from Tax1BP1^+/+^ as well as Tax1BP1^−/−^ mice. Tax1BP1^−/−^ mice showed a reduced hepatic expression of IκBα at basal conditions compared to Tax1BP1^+/+^ mice (Fig. 3A). Moreover, hepatocytes from Tax1BP1^−/−^ mice showed higher IκBα degradation following TNFα stimulation compared to hepatocytes from Tax1BP1^+/+^ mice at the indicated time points (Fig. 3A). TNFα led to increased NFκB-p65 phosphorylation and to a more profound decrease in p65 levels from to the cytoplasmatic fraction upon stimulation in Tax1BP1^−/−^ mice compared to Tax1BP1^+/+^ mice, respectively (Fig. 3B). Furthermore, hepatocytes from Tax1BP1^−/−^ mice tended to show a faint increase in JNK activation following TNFα challenge compared to hepatocytes from Tax1BP1^+/+^ mice (Fig. 3A).

### Differentially regulated genes in livers of Tax1BP1^−/−^ mice

Tax1BP1 is a regulator of proinflammatory and stress-activated signaling pathways. As there is only little data of the role of Tax1BP1 in regulating hepatic expression of mRNA and miRNA levels, microarray analyses in liver tissue from Tax1BP1^+/+^ as well as Tax1BP1^−/−^ mice were performed (Fig. 3C,D). Livers of Tax1BP1^−/−^ mice showed increased expression of genes involved in immune cell regulation (BCL6, CXCL13, KLF10, Lysozyme 2), cell cycle control, differentiation, and senescence (OSGIN1, Hist1h2ao, Hes1, Tbx3, Efna1, Id2) (Fig. 3C, Table 1). In contrast genes involved in development and differentiation (Arid5b, Inmt, Grem2, Gdap10), cell metabolism (Alas1, Gsta1, Thrsp, Fdft1, Elovl6), tumor growth (SerpinA4, Cish, Rpl36), and cell cycle control (PLK3, Myc, HNF6/Onecut1) were downregulated (Fig. 3C, Table 1). In addition, we compared the expression of miRNAs between Tax1BP1^+/+^ and Tax1BP1^−/−^ mice. miR-31 and miR-192 were less expressed in livers of Tax1BP1^−/−^ mice. Both miRNAs have been shown to limit inflammation and to restrain tumor growth, whereas the oncogenic miR-92, which promotes inflammation and tumor progression was overexpressed in Tax1BP1^−/−^ mice (Fig. 3D, Table 2).

**Table 1:** Differentially expressed mRNA in Tax1BP1^−/−^ mice.

Name	mRNA Accession	x-fold expression	*P* value
**Upregulated genes**			
Bcl6	NM_009744	3.0	0.017
Cxcl13	NM_018866	2.2	< 0.0001
Osgin1	NM_027950	2.2	0.034
Klf10	NM_013692	1.9	< 0.001
Hist1h2ao	NM_001177544	1.8	0.010
Lysozyme 2	NM_017372	1.7	0.007
Hes1	NM_008235	1.6	0.033
Tbx3	NM_011535	1.6	0.042
Atp11a	NM_015804	1.5	0.013
Efna1	NM_010107	1.5	0.021
Id2	NM_010496	1.5	0.016
**Downregulated genes**			
Alas1	NM_020559	3.3	< 0.001
Gsta1	NM_008181	3.1	0.035
Plk3	NM_013807	2.8	0.001
Serpina4-ps1	BC031891	2.6	0.049
Myc	NM_010849	2.5	0.037
Thrsp	NM_009381	2.2	0.006
Onecut1	NM_008262	2.1	0.020
Arid5b	NM_023598	1.9	0.017
Tax1bp1	NM_025816	1.9	0.003
Cish	NM_009895	1.9	0.004
Inmt	NM_009349	1.6	0.012
Rangrf	NM_021329	1.6	0.034
Grem2	NM_011825	1.6	0.010
Gdap10	BC052902	1.6	0.039
Rpl36	NM_018730	1.5	0.026
Fdft1	NM_010191	1.5	0.033
Elovl6	NM_130450	1.5	0.011

**Table 2:** Differentially expressed miRNA in Tax1BP1^−/−^ mice.

Name	x-fold expression	*P* value
**Upregulated miRNAs**		
hp_mmu-mir-5119_st	2.4	0.004
mmu-miR-714_st	2.2	0.001
hp_mmu-mir-3093_st	2.0	0.018
hp_mmu-mir-5098_x_st	2.0	0.038
mmu-miR-1934-star_st	2.0	0.015
mmu-miR-346-star_st	2.0	0.049
mmu-miR-3102-star_st	1.9	0.006
mmu-miR-5119_st	1.8	0.035
hp_mmu-mir-3473_st	1.7	0.032
mmu-miR-3470b_st	1.7	0.010
mmu-miR-5102_st	1.7	0.029
mmu-miR-5115_st	1.7	0.014
mmu-miR-712_st	1.7	0.041
mmu-miR-696_st	1.6	0.027
mmu-miR-5105_st	1.5	0.030
mmu-miR-5135_st	1.5	0.019
mmu-miR-705_st	1.5	0.011
mmu-miR-92b-star_st	1.5	0.030
**Downregulated miRNAs**		
mmu-miR-31-star_st	1.5	0.004
mmu-miR-195_st	1.5	0.024
mmu-miR-125a-5p_st	1.5	0.033

#### CCl_4_ induced liver fibrosis

**Figure 4:**
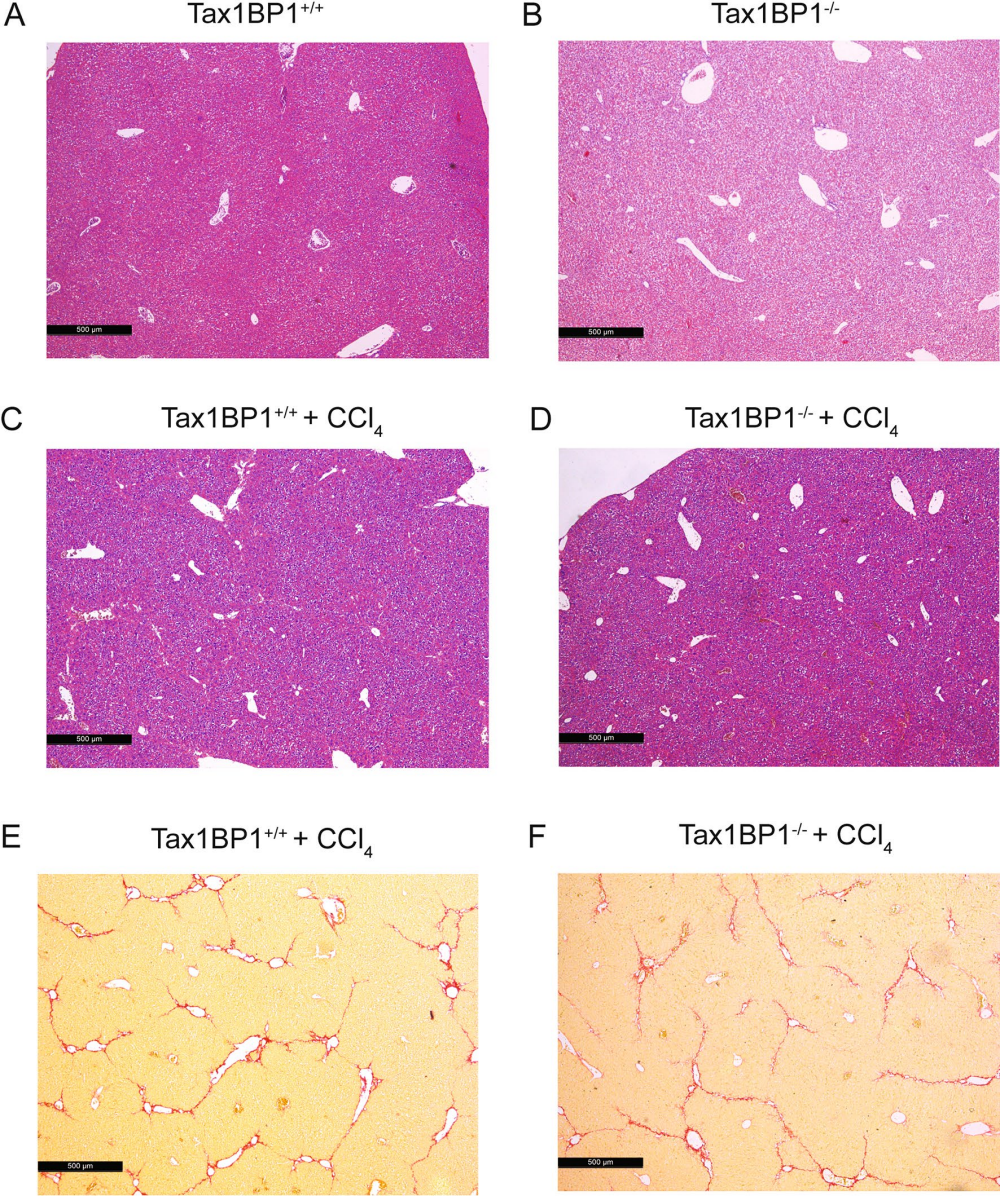
CCl_4_ induces liver fibrosis in Tax1BP1^+/+^ and Tax1BP1^−/−^ mice. Liver sections of Tax1BP1^+/+^ and Tax1BP1^−/−^ mice before and after induction of liver fibrosis with CCl_4_ were stained with hematoxylin and eosin (**A**–**D**). Liver sections of CCl_4_ treated Tax1BP1^+/+^ and Tax1BP1^−/−^ mice were stained with Sirius red (**E**,**F**). Scale bars are included in the individual pictures, respectively.

As Tax1BP1^−/−^ mice showed an inflammatory phenotype, we deduced that experimental fibrosis development might be affected by Tax1BP1. Therefore, fibrosis was induced in Tax1BP1^+/+^ mice as well as in their Tax1BP1^−/−^ littermates by CCl_4_ treatment. In each group eight mice were used with 50% of female mice. Both mouse strains developed fibrosis after CCl_4_ treatment. There was no difference in the stage of fibrosis between Tax1BP1^−/−^ mice and the Tax1BP1^+/+^ mice seven weeks after start of fibrosis induction, respectively (Fig. 4C–F). Exemplary untreated mouse tissue sections of livers of Tax1BP1^+/+^ and Tax1BP1^−/−^ and mice are displayed in Fig. 4A,B.

#### DEN induced hepatocellular carcinoma

**Figure 5:**
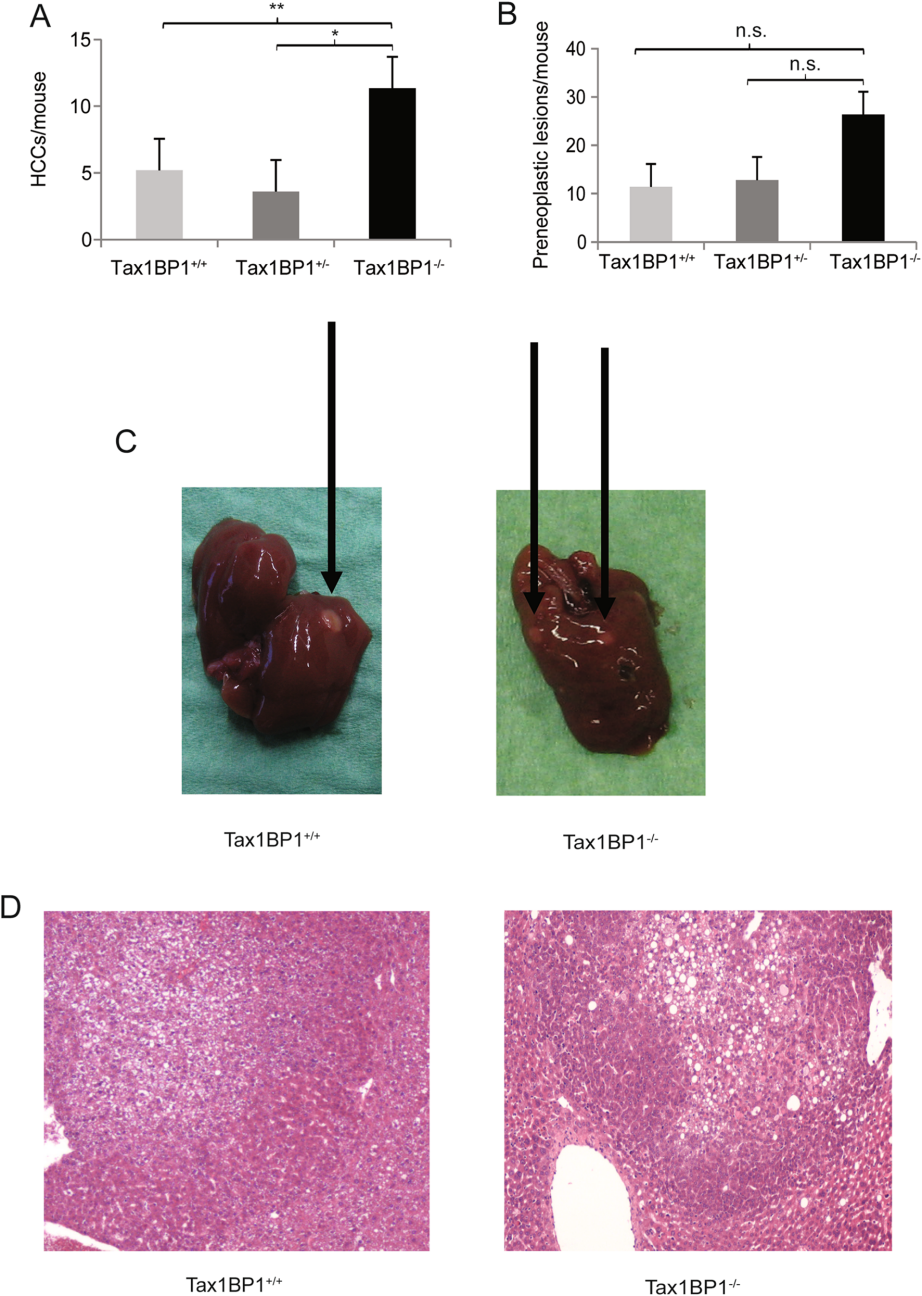
Liver tumor development. Number of hepatocellular carcinomas (**A**) and pre-neoplastic tumors (**B**) in male Tax1BP1^+/+^ (n = 5), Tax1BP1^+/−^ (n = 5) and Tax1BP1^−/−^ (n = 3) mice after DEN treatment are shown in bar graphs. Differences in the number of tumors between different strains were determined using the nonparametric Kruskal–Wallis test *P < 0.05; **P < 0.01; *n.s.* not significant). Representative macroscopic livers (**C**) and representative histological hematoxylin and eosin stained tumor slides (**D**) from Tax1BP1^+/+^ and Tax1BP1^−/−^ mice are shown.

The NFκB signaling pathway plays an important role in liver homeostasis and inflammation. Dependent on its activity in the different hepatic cell compartments, it may limit tumor formation but it can also promote carcinogenesis. As Tax1BP1 was expressed in the different hepatic cell types, we concluded that it could be an important factor in hepatic carcinogenesis. To investigate if Tax1BP1 might modulate liver cancer development, hepatocarcinogenesis was induced in Tax1BP1^+/+^, Tax1BP1^+/−^ and Tax1BP1^−/−^ mice using DEN treatment. After eight months, the livers were removed and the number of HCCs and preneoplastic tumors including dysplastic foci and nodules was assessed by a pathologist in a blinded manner. Tax1BP1^+/+^, Tax1BP1^+/−^ as well as Tax1BP1^−/−^ developed pre-neoplastic tumors and HCCs. There were significant differences among Tax1BP1^+/+^, Tax1BP1^+/−^ and Tax1BP1^−/−^ mice in the number of HCCs with Tax1BP1^−/−^ mice showing significantly more HCCs (Fig. 5A). Tax1BP1^−/−^ mice also developed more preneoplastic lesions compared to their Tax1BP1^+/+^ and Tax1BP1^+/−^ littermates. However, this difference was not statistically significant (Fig. 5B). Representative macroscopic and microscopic images for Tax1BP1^+/+^and Tax1BP1^−/−^ mice are shown (Fig. 5C,D).

## Discussion

The equilibrium between cell death and survival is decisive for organ homeostasis. Dysregulations of both ways may be fatal leading either to massive cell death, cancer development or both^6,9^. The NFκB signaling pathway is a central regulator of cell death and survival and its effects on cells and tissues are highly dependent on the cell type^24,25^. In the DEN model of experimental HCC, NFκB activity in hepatocytes protects from cancer development, whereas the activation of this pathway in the bone marrow derived cells promotes cancer development and progression^6–8^.

Tax1BP1 is highly expressed in myeloid derived cells and the expression of Tax1BP1 in bone marrow derived cells determines its phenotype as NFκB inhibiting protein in an experimental model mimicking sepsis^15^, and Tax1BP1^−/−^ mice show a spontaneous hepatic inflammatory phenotype^17^. Here we found an increased number of cells of the innate and adaptive immune system in the livers of Tax1BP1^−/−^ mice and an enhanced expression of proinflammatory cytokines such IL1β and TNFα in isolated Kupffer cells derived from Tax1BP1^−/−^ mice after LPS stimulation. This supports the observation that the livers of Tax1BP1^−/−^ mice show an inflammatory phenotype. Nevertheless, we further show that Tax1BP1 is not only expressed in non-parenchymal hepatic cells but also in hepatocytes and that loss of Tax1BP1 expression was associated with a stronger activation of the NFκB signaling in isolated hepatocytes. Interestingly, we also found a slightly increased SAPK/JNK activity in hepatocytes from Tax1BP1^−/−^ mice in comparison to their wildtype littermates after TNFα challenge. The JNK pathway plays a critical role in liver disease, and hyperactivation or loss of the pathway may influence inflammation and cancer development^26^. Two different Tax1BP1 knock-out mouse models revealed differences concerning the activation of the SAPK/JNK pathway^15,27^. In our current mouse model no relevant increase in JNK activation was observed in macrophages in contrast to another Tax1BP1 knock-out model^15,27^. Namely, we found a slight increase in JNK phosphorylation in hepatocytes from Tax1BP1^−/−^ mice, indicating that the extent of affection of this pathway by Tax1BP1 might be cell-type dependent.

Nonetheless, Tax1BP1 seems to play its major role in immune cells, as we found an increase of cells of the innate as well as adaptive immune systems in Tax1BP1^−/−^ mice supporting the role of Tax1BP1 as an important regulator of inflammation. Furthermore, it has recently been shown that Tax1BP1 is not only an adaptor for deubiquitinating enzymes such as A20^15^, but it also affects adaptive immunity including B and T cells by regulating autophagy^28,29^. Tax1BP1^−/−^ mice exhibited increased levels of lymphocytes and especially CD8 positive T cells and regulatory T cells were significantly augmented. The increase in the number of cells of the innate and adaptive immunity in livers Tax1BP1^−/−^ mice of came along increased expression of regulators of inflammation and immune cell differentiation such as BCL6, CXCL13, KLF10, and Lysozyme 2 in Tax1BP1^−/−^ mice in array based analyses from liver tissue. miRNAs are epigenetic regulators of gene expression. In our array based miRNA analyses we found several miRNAs up- or downregulated in Tax1BP1^−/−^ mice. Some of these are well known as regulators of inflammation or are involved in cancer development or progression. Namely, miRNAs such as miR-31 and miR-192 which limit inflammation and cancer development^30–32^, were downregulated and miR-92, which increases liver tumor development^33^ was overexpressed in Tax1BP1^−/−^ mice. Interestingly, we did not observe increase fibrosis development in Tax1BP1^−/−^ mice with the CCL_4_ model we used. This was unexpected as NFκB activiation is associated with inflammation and fibrosis development^24^. Therefore, additional models of fibrosis such as bile duct ligation and other concentrations of CCl_4_ might be used to further decipher the effect of Tax1BP1 in fibrosis development.

Increased NFκB activation is an early event in HCC development^34^. However, the role of NFκB as a promotor or an inhibitor seems to be rather cell type specific^24^. In transformed hepatocytes, inhibition of NFκB signaling attenuates tumor progression^6^, whereas ablation of central NFκB signaling pathways leads to spontaneous HCC development^7^, or may trigger JNK activation, which promotes carcinogenesis^35^. Our Tax1BP1^−/−^ mice showed an increased activation of the NFκB signaling pathway in hepatocytes as well as increased numbers of inflammatory cells leading to a spontaneous inflammatory phenotype in the liver. Inhibition of NFκB signaling by ablation of IKKβ in myeloid derived cells reduces HCC development in mice^8^. Therefore, our observation of an increased development of HCCs in Tax1BP1^−/−^ mice with increased numbers of non-parenchymal liver cells and increased levels of proinflammatory cytokines upon stimulation of macrophages fits in the theory of a tumor promoting effect of NFκB in myeloid derived cells by enhancing anti-apoptotic signaling in hepatocytes triggered by Kupffer and hepatic stellate cells predisposes transformed cells to cancer development^3–5^.

Another interesting aspect of Tax1BP1 is that Tax1BP1 has been described as a regulator of antiviral signaling. Together with A20, it targets the TBK1-IKKi kinases and restricts INFβ production^36^. IKKi/IKKepsilon has an important antiviral function in chronic hepatitis C virus infection, as it limits viral replication and itself is down regulated in infected cells^37^. Furthermore enhanced genomic amplification of Tax1BP1 has been described in a subset of hepatocellular carcinomas, which developed in patients with hepatitis C virus infection^18^. Therefore, further investigation of Tax1BP1 in chronic liver disease may be of great interest and may open new vistas on this protein and its role in diseases.

## Supplementary information

Supplementary Information.

## Abbreviations

NFκB: Nuclear factor kappa beta
Tax1BP1: Tax1-binding protein 1
HCC: Hepatocellular carcinoma
DEN: Dimethylnitrosamine
FACS: Fluorescence-activated cell sorting
HBV: Hepatitis B virus
HCV: Hepatitis C virus
MAPK: Mitogen-activated protein kinases
mTOR: Mammalian target of rapamycin
IKK β: IκB kinase β
IKK: IκB kinase
TNFα: Tumor necrosis factor α
LPS: Lipopolysaccharides
IL-1β: Interleukin-1β
HBSS: Hank’s buffered saline
FCS: Fetal calf serum
PBS: Phosphate buffered saline
MACS: Magnetic activated cell sorting
BSA: Bovine serum albumin
