# Supplementary material for: Tax1BP1 limits hepatic inflammation and reduces experimental hepatocarcinogenesis
Source: Sci Rep. 2020 Oct 1;10:16264. doi: 10.1038/s41598-020-73387-4 (PMC7530720; doi:10.1038/s41598-020-73387-4)

**Tax1BP1 limits hepatic inflammation and reduces experimental hepatocarcinogenesis**

Oliver Waidmann^1,2^, Thomas Pleli^1^, Andreas Weigert^3^, Esther Imelmann^1^, Bianca Kakoschky^1^, Claudia Döring^4^, Matthias Frank^4^, Thomas Longerich^5^, Verena Köberle^1^, Martin-Leo Hansmann^4^, Bernhard Brüne^3^, Stefan Zeuzem^1^, Albrecht Piiper^1^, and Ivan Dikic^2^

^1^Medizinische Klinik 1, Schwerpunkt Gastroenterologie und Hepatologie, Universitätsklinikum Frankfurt, Goethe-Universität, Theodor-Stern-Kai 7, D-60590 Frankfurt, Germany

^2^Institut für Biochemie 2, Universitätsklinikum Frankfurt, Goethe-Universität, Theodor-Stern-Kai 7, D-60590 Frankfurt, Germany

^3^Institut für Biochemie 1, Universitätsklinikum Frankfurt, Goethe-Universität, Theodor-Stern-Kai 7, D-60590 Frankfurt, Germany

^4^Senckenbergsches Institut für Pathologie, Universitätsklinikum Frankfurt, Goethe-Universität, Theodor-Stern-Kai 7, D-60590 Frankfurt, Germany

^5^Sektion Translationale Gastrointestinale Pathologie, Institut für Pathologie, Universitätsklinikum Heidelberg, Im Neuenheimer Feld 224, 69120 Heidelberg, Germany

Gene Arrays:

Total RNA was extracted from mouse liver specimens using the RNeasy Mini Kit (Qiagen, Hilden, Germany) following the manufacturers protocol. RNA quantification was performed using a Nanodrop 2000 spectrometer (Thermo Fisher Scientific, Wilmington, DE, USA) and an Agilent 2100 Bioanalyzer instrument (Agilent Technologies) was used for quality control.

The total RNA (input: 50ng) amplification and cDNA labelling was done using standardized protocols (Ovation Pico WTA System V2 amplification kit and Encore Biotin Module labelling kit from NuGEN). Microarray hybridization to GeneChip Mouse Gene 1.0 ST V1 arrays (Affymetrix), washing steps, and scanning of the microarrays were performed according to the Affymetrix protocol.

The data discussed in this publication have been deposited in the NCBI Gene Expression Omnibus and are accessible through GEO series accession number GSE98908.

miRNA Arrays:

Total RNA was extracted from mouse liver specimens using the miRNeasy Mini Kit (Qiagen, Hilden, Germany) following the manufacturers protocol. RNA quantification was performed using a Nanodrop 2000 spectrometer (Thermo Fisher Scientific, Wilmington, DE, USA) using 500 ng of total RNA for further analyses.

A global miRNA expression profiling analysis of 1111 mature miRNAs using Genechip miRNA array v3.0 (Affymetrix, Santa Clara, CA, USA) was performed following the manufacturer's protocol. The data discussed in this publication have been deposited in the NCBI Gene Expression Omnibus and are accessible through GEO series accession number GSE98907.

Statistical Analysis

The statistical analysis was done with the statistical computing environment R version 2.12 [38]. Additional software packages were taken from the Bioconductor project [39].

Microarray Preprocessing

Normalisation

Probe level normalization was conducted using the variance stabilization method by [40]. This method renders the variance of probe intensities approximately independent of their expected expression levels. Parameters (offset and a scaling factor) are estimated for each microarray, in consideration of the fact that a fair fraction of probes is not differentially expressed across the samples. In view of the computational complexity of the algorithm parameters are estimated on a random subset of probes and are then used to transform the complete arrays.

Probeset summary

Probeset summarization was calculated using the median polish method [41] on the normalized data.

For each probeset a robust additive model was fitted across the arrays, considering the different sensitivity of the probesets via the probe effect.

Differentially expression

Many of the genes on the microarray won’t be expressed, or might have only a small variability across the samples. First we use an expression intensity filter to reduce the dimension of the microarray data: We filter the data with an intensity filter (the intensity of a gene should be above 100 in at least 0.25 percent of the samples, if the group size is equal) and a variance filter (the interquartile range of log2 intensities should be at least 0.5, if the group size is equal).

After the expression intensity filtering we calculate p- values with the two sample t-test (variance=equal) to identify genes that are differentially expressed between two groups. For the multiple testing problems we use a False Discovery Rate (FDR) [42]. Also Fold Changes (FC) between the two groups was calculated for each gene. The lists of differentially expressed genes were filtered with FDR and FC criteria.

Isolation and characterization of hepatic leucocytes by flow cytometry:


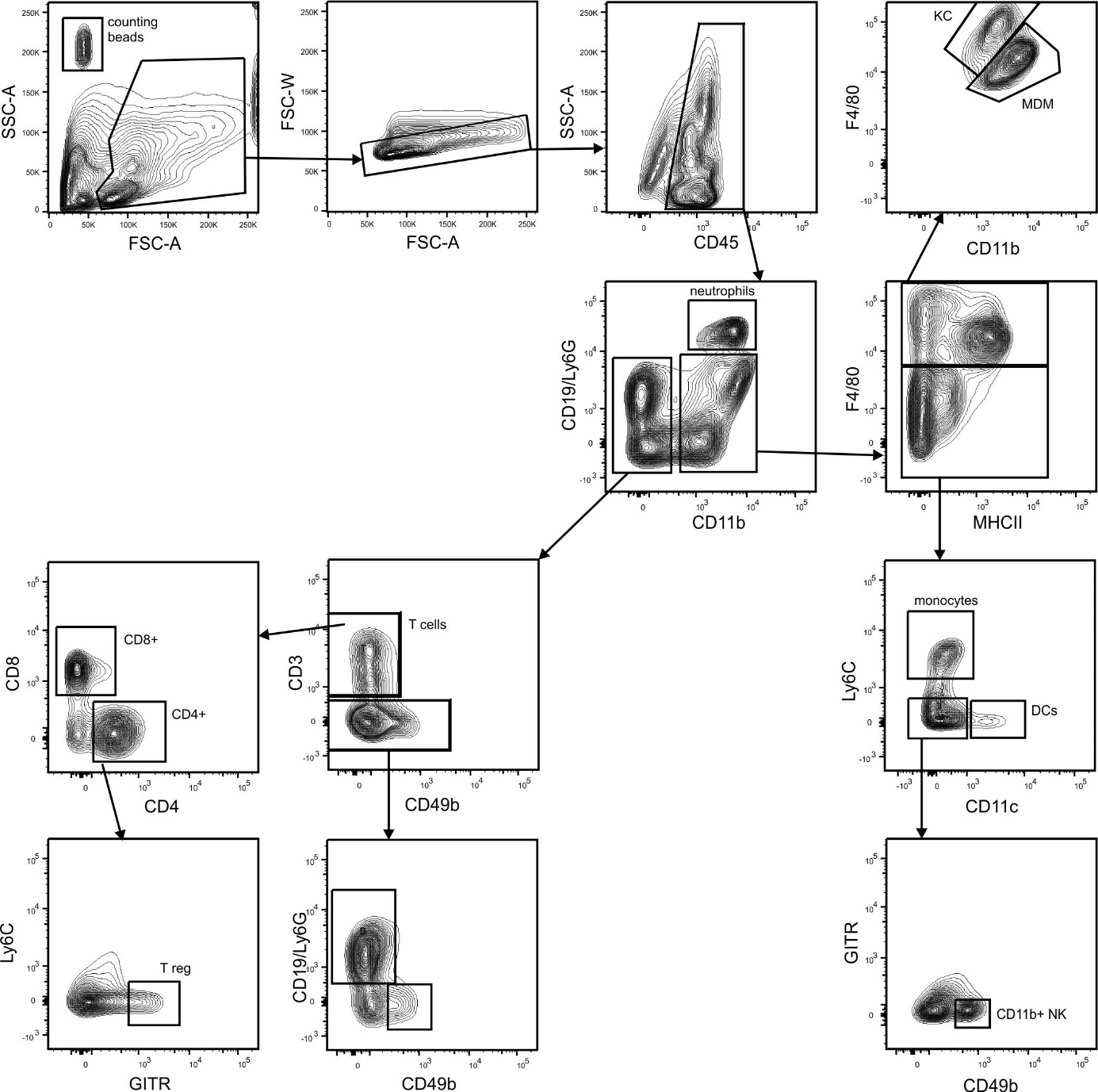


Supplementary Figure 1: Exemplary gating strategy to define liver immune cell subpopulations.

References:

[38] R Development Core Team.R: A language and environment for statistical computing. R Foundation for Statistical Computing, Vienna, Austria, 2005. URL http://www.R-project.org.ISBN 3-900051-07-0.

[39] Gentleman, R.C., Carey, V.J., Bates, D.M., Bolstad, .B, Dettling, M., Dudoit, S., et al. Bioconductor: Open software development for computational biology and bioinformatics. *Genome Biol.* **5,** R80 (2004).

[40] Huber, W., von Heydebreck, A., Sültmann, H., Poustka, A., Vingron, M. Variance stabilization applied to microarray data calibration and to the quantification of differential expression. *Bioinformatics*, 18(Suppl 1):96-104, 2002. ISSN 1367-4803.

[41] Tukey, JW. Exploratory Data Analysis. Addison-Wesley, Reading Masschusetts, USA, 1977.

[42] Hochberg, Y., Benjamini, Y. More powerful procedures for multiple significance testing. *Stat Med.* **9**, 811-818 (1990).

Supplementary data – Western Blots

Tax1BP1 + Actin control


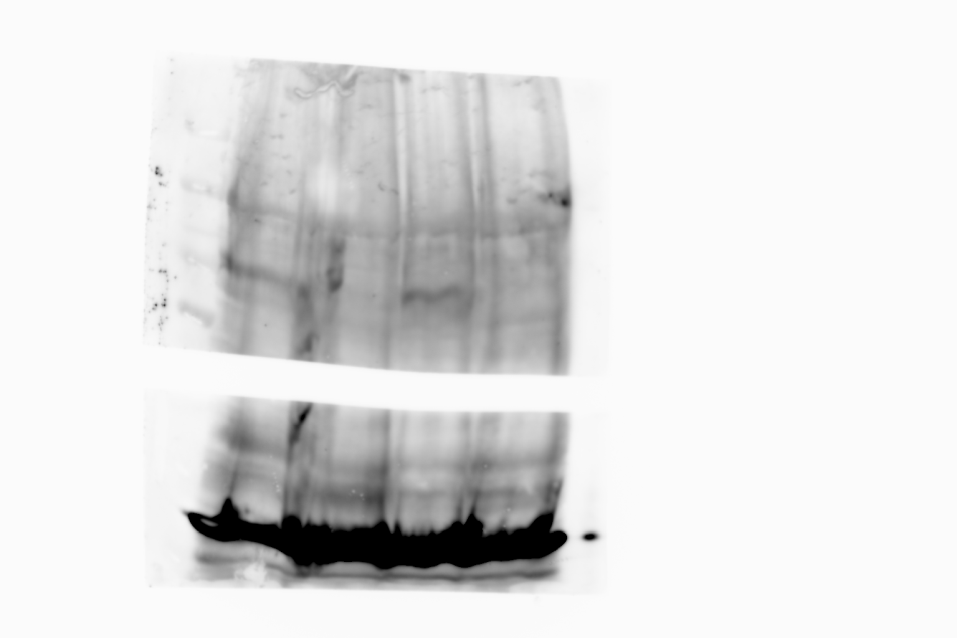


Actin control short exposure


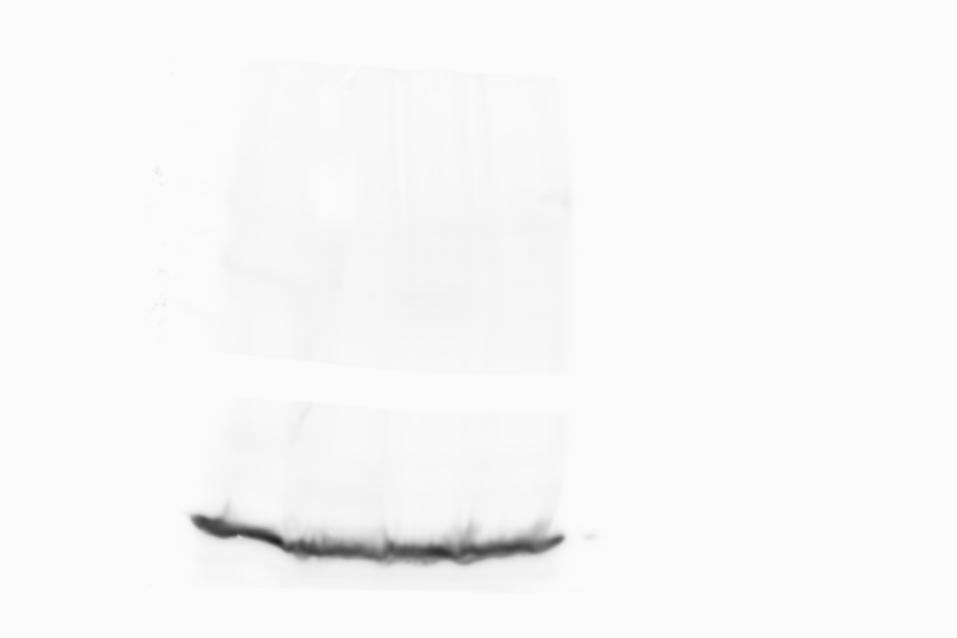


pJNK


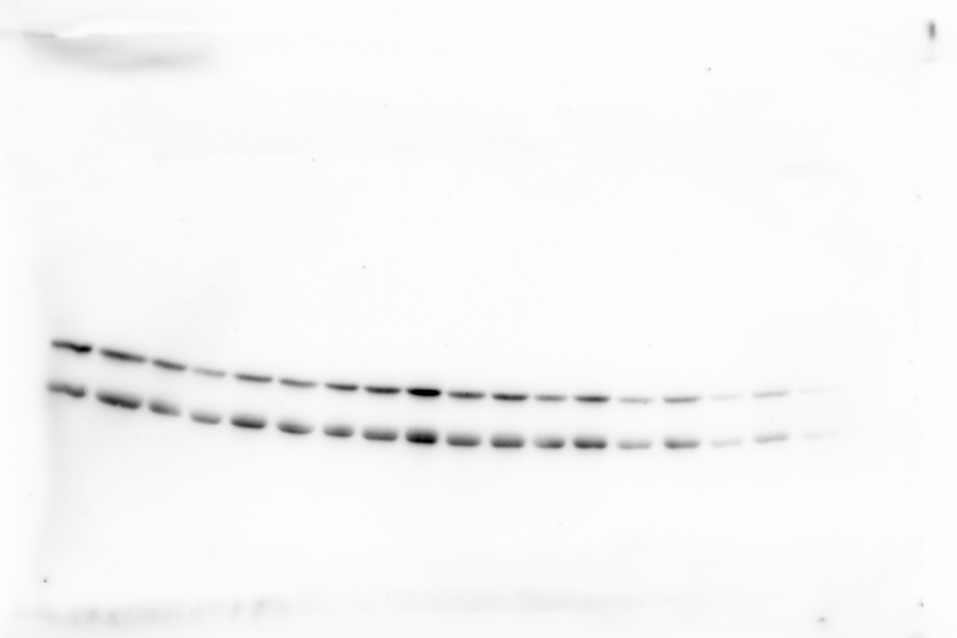


Total JNK


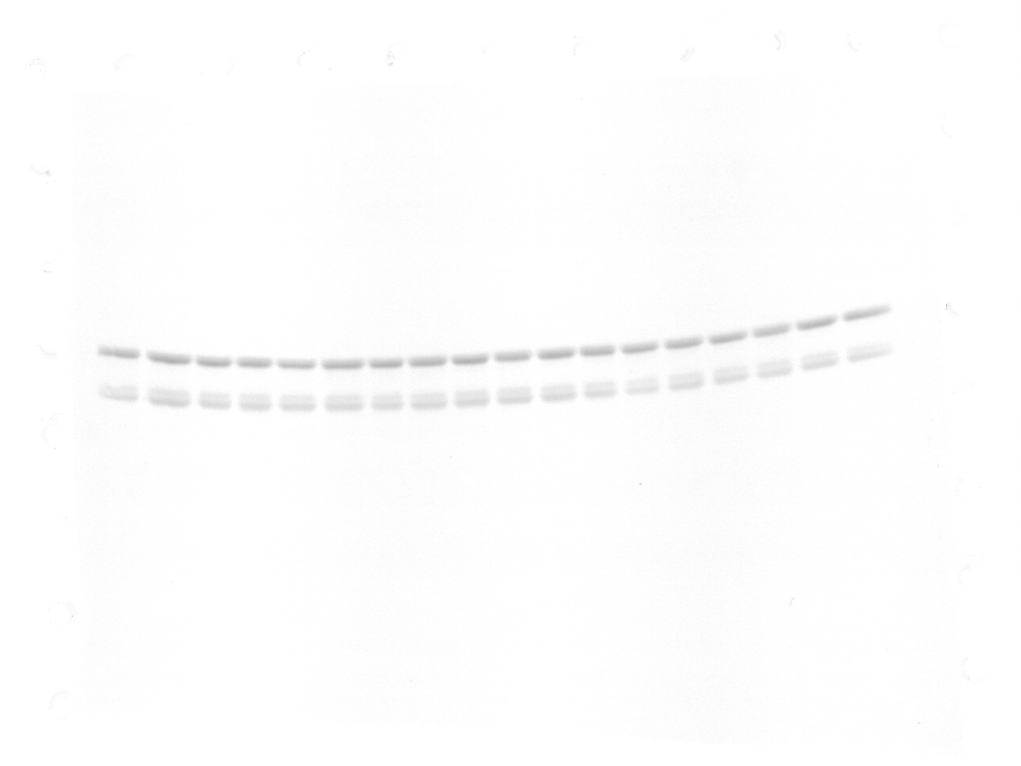


IKba


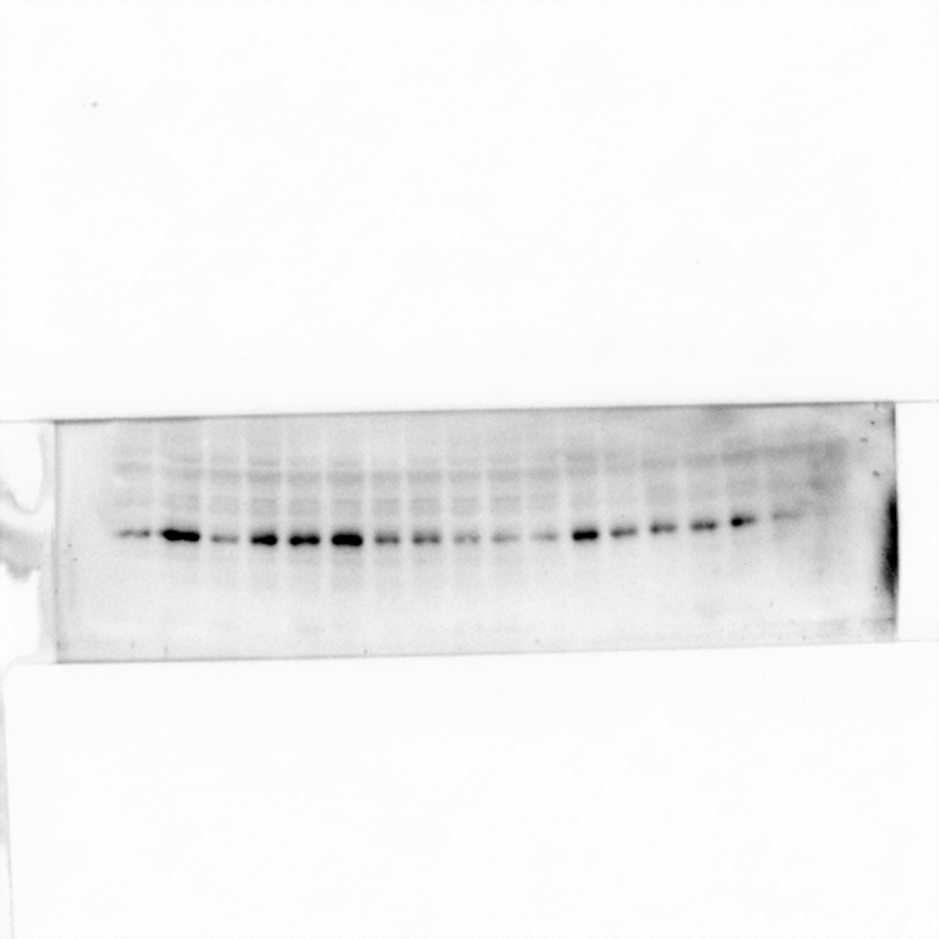


Actin


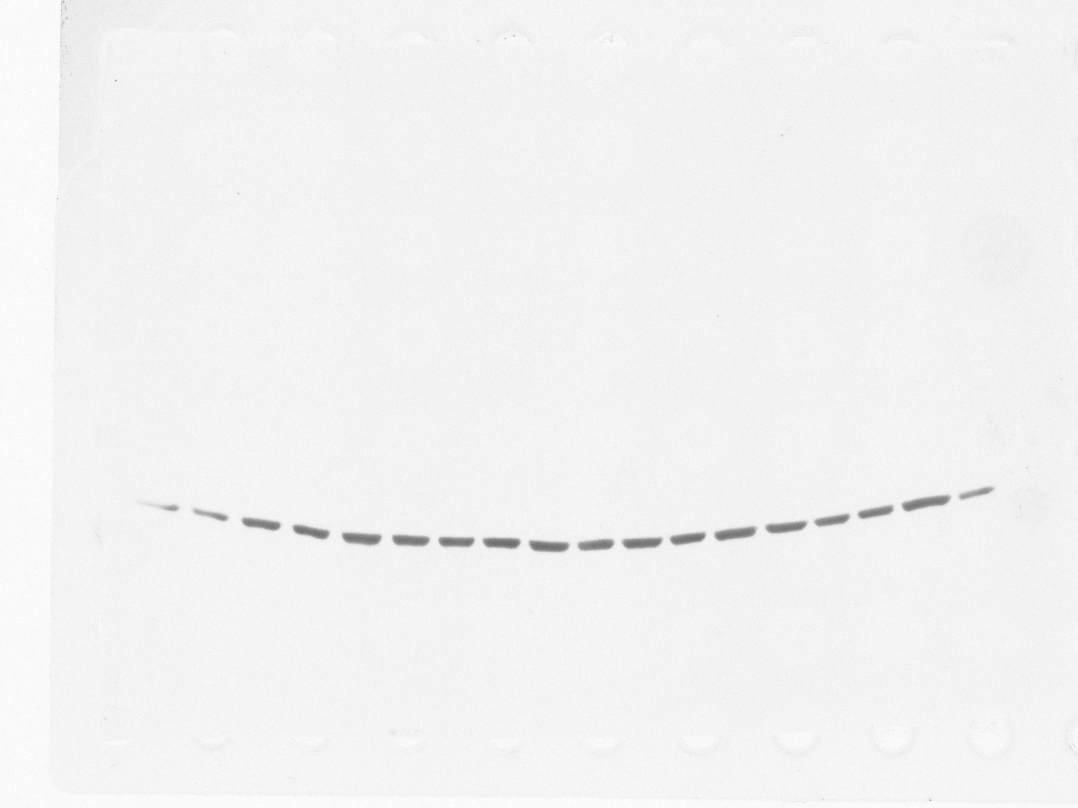


Phospho-NFKB (p65)


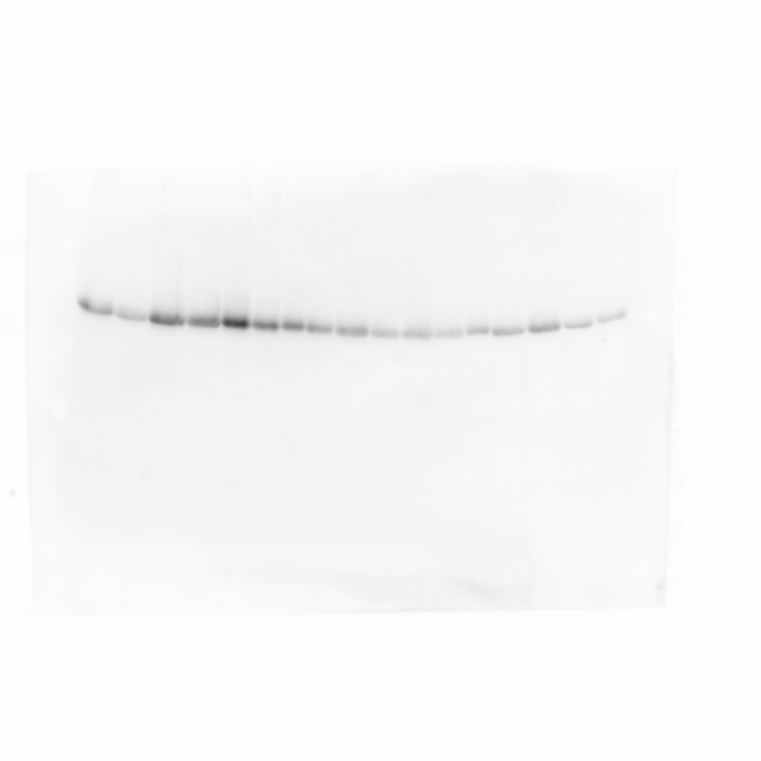


Total NFKB (p65)


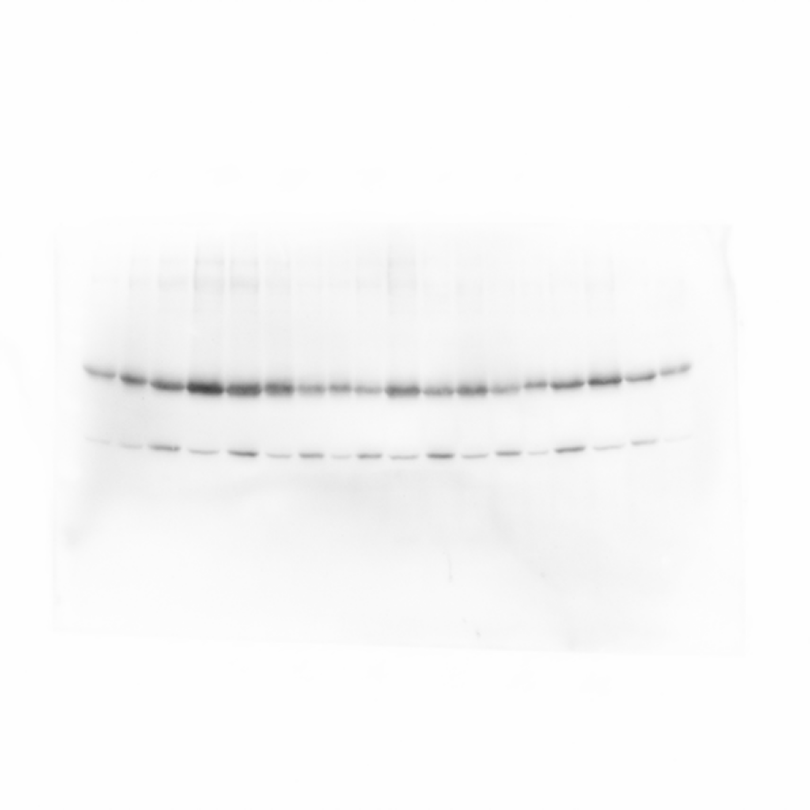


total JNK control for pp65


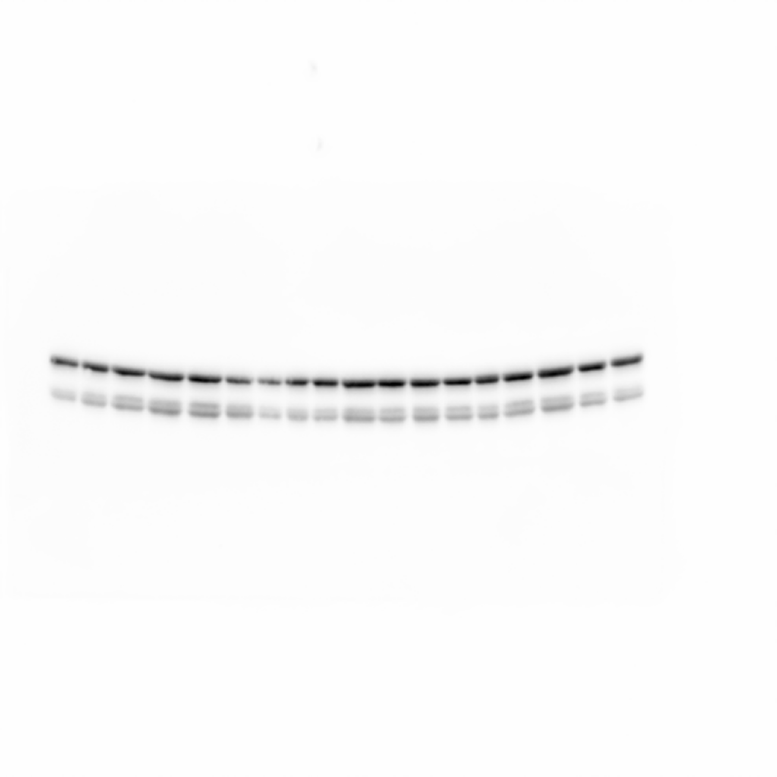

Supplement: Supplementary file 1 — Supplementary Information. [file 41598_2020_73387_MOESM1_ESM.docx]
